# Antithrombotic Therapy in Percutaneous Atrial Structural Interventions

**DOI:** 10.3390/jcdd13030108

**Published:** 2026-02-26

**Authors:** Konstantinos Pitsikakis, Ioannis Skalidis, Emmanuel Skalidis, Dimitrios Lempidakis, Antonios Papoutsakis, Emmanuel Sideras, Evangelos Zacharis, Stylianos Petousis, Michalis Hamilos

**Affiliations:** 1 Department of Cardiology, University Hospital of Heraklion, Voutes, Leof Panepistimiou, 71500 Iraklio, Greece; kpitsika@uoa.gr (K.P.); skalides@uoc.gr (E.S.); dimlemp@hotmail.com (D.L.); antgeopap@yahoo.gr (A.P.); manolis.1994@windowslive.com (E.S.); ezacharis@yahoo.gr (E.Z.); sgpetousis@gmail.com (S.P.); 2 Department of Cardiology, HFR—Fribourg Hospital and University, 1708 Fribourg, Switzerland; skalidis7@gmail.com

**Keywords:** left atrial appendage occlusion, patent foramen ovale closure, atrial septal defect closure, postprocedural antithrombotic strategies, antiplatelet therapy, anticoagulant therapy

## Abstract

Percutaneous left atrial appendage occlusion (LAAO), patent foramen ovale (PFO) closure, and atrial septal defect (ASD) closure rely on temporary antithrombotic therapy to prevent device-related thrombus during endothelialization, yet optimal regimens remain uncertain and vary widely across clinical practice. This review synthesizes contemporary evidence on postprocedural antithrombotic strategies, comparing efficacy and safety data and identifying key gaps in knowledge. After LAAO, therapeutic approaches range from short-term anticoagulation with vitamin K antagonists or direct oral anticoagulants to dual or single antiplatelet therapy in patients with high bleeding risk; observational data increasingly support DOAC-based regimens, although device-related thrombus remains a significant concern, and follow-up imaging protocols are inconsistent. Following PFO and ASD closure, antiplatelet-only regimens—typically brief dual antiplatelet therapy followed by aspirin—are widely used, with evidence suggesting that simplified or abbreviated strategies may be sufficient in selected patients. Despite extensive clinical experience, high-quality comparative trials are limited, and optimal therapy, duration, and surveillance remain debated. Standardized imaging definitions, randomized studies, and individualized risk-based frameworks are needed to optimize antithrombotic care after atrial structural interventions.

## 1. Introduction

Transcatheter interventions for structural heart disease, such as left atrial appendage (LAA) occlusion, patent foramen ovale (PFO) closure, and secundum atrial septal defect (ASD) closure, remain fundamental therapies in modern cardiology [1,2]. These procedures aim to mitigate stroke risk and correct hemodynamic disturbances associated with intracardiac shunts, achieving high procedural success with favorable safety profiles. Recent advances in device technology and procedural techniques further reinforce their role in contemporary practice [3].

Nevertheless, there is no universal consensus on the optimal postprocedural antithrombotic regimen [4]. Clinical practice varies considerably regarding the type (e.g., dual antiplatelet therapy [DAPT], single antiplatelet therapy [SAPT], direct oral anticoagulants [DOACs]), the duration of therapy, and the balance between ischemic and bleeding risks [3,5,6,7]. Current expert consensus statements highlight the lack of robust randomized data to guide uniform treatment paths, especially in high-bleeding-risk patients after left atrial appendage closure (LAAC) [8,9]. Moreover, as patient populations broaden—including older adults, those with prior bleeding, or with comorbidities—the need to tailor antithrombotic strategies becomes more demanding. For instance, recent real-world data suggest that even shortened antithrombotic regimens (e.g., ≤6 months) may be feasible in selected high-risk cohorts [10,11]. In parallel, an evolving body of literature examines the long-term outcomes, device-related thrombosis, and safety implications of different therapeutic strategies [12,13,14].

In this context, the present systematic review seeks to synthesize current evidence on antithrombotic therapy following LAA, PFO, and ASD closure. Our goals are to map prevailing clinical practices, compare efficacy and safety among regimens, and identify critical knowledge gaps that should inform both clinical care and future research.

## 2. Materials and Methods

A literature review was conducted using the PubMed/MEDLINE database to identify studies relevant to post-procedural antithrombotic therapy following left atrial appendage occlusion (LAAO), patent foramen ovale (PFO) closure, and atrial septal defect (ASD) closure. The search covered publications from January 2004 through January 2026, with the majority of included studies published from 2020 onward, reflecting the rapid evolution of contemporary device technology and antithrombotic strategies.

The following Boolean search strategy was applied: (“left atrial appendage occlusion” OR “LAAO” OR “left atrial appendage closure”) AND (“antithrombotic therapy” OR “antiplatelet” OR “anticoagulation” OR “DOAC” OR “dual antiplatelet therapy”) AND (“patent foramen ovale closure” OR “PFO closure” OR “atrial septal defect closure” OR “ASD closure”).

Eligible studies included randomized controlled trials, prospective and retrospective cohort studies, registries, and systematic reviews reporting clinical outcomes related to device-related thrombus, thromboembolism, stroke, bleeding, or mortality after structural atrial interventions. Case reports and small case series were included only when addressing rare but clinically relevant complications. Studies were limited to English-language publications. Grey literature, including conference abstracts without full-text publication, preprints, and non-peer-reviewed sources, was not included.

## 3. Antithrombotic Therapy After LAAO

### 3.1. Background

Current ESC guidelines specify that percutaneous left atrial appendage occlusion may be considered (Class IIb, level C) to prevent stroke in patients with non-valvular atrial fibrillation at high thromboembolic risk who are not suitable for long-term oral anticoagulation, but emphasize the limited evidence base and ongoing uncertainty regarding optimal antithrombotic strategies after device implantation [15,16,17]. The early period following implantation is characterized by incomplete endothelialization of the device surface, which creates a transient prothrombotic state that may lead to device-related thrombus (DRT) [18]. Because DRT is associated with an increased risk of thromboembolic complications, short-term antithrombotic therapy is routinely employed to mitigate this risk. Despite this common objective, post-procedural regimens vary widely among institutions, largely due to the absence of solid randomized evidence [19,20,21,22,23].

### 3.2. Available Therapeutic Strategies

**Figure 1:**
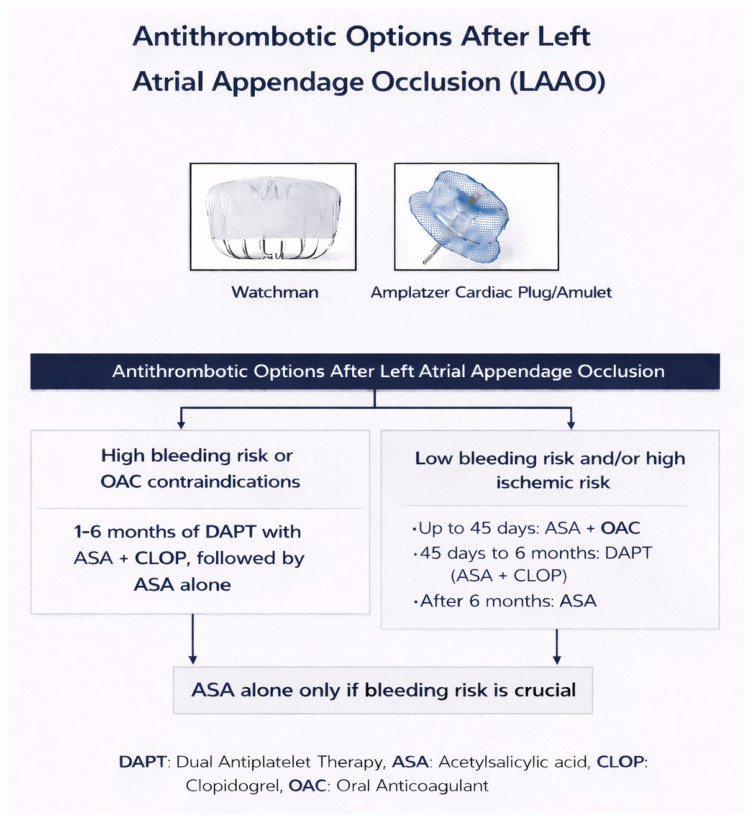
Treatment algorithm for antithrombotic therapy after LAAO. DAPT (Dual Antiplatelet Therapy), ASA (Acetylsalicylic Acid), CLOP (Clopidogrel), OAC (Oral Anticoagulant) [TITLE: Antithrombotic Options After Left Atrial Appendage Occlusion (LAAO)].

Clinical practice includes several distinct antithrombotic approaches. Many centers initiate short-duration oral anticoagulation (OAC)—either vitamin K antagonists or direct oral anticoagulants (DOACs)—for the first 45 days to 3 months, often guided by follow-up imaging to confirm adequate healing and the absence of thrombus. In cases where anticoagulation is considered unsafe, operators may use dual antiplatelet therapy (DAPT) for a short period. Single antiplatelet therapy (SAPT) is generally reserved for patients with exceptionally high bleeding risk. Increasingly, DOAC monotherapy is also being adopted based on emerging observational data (Figure 1). The coexistence of these strategies reflects the lack of a universally accepted regimen [15,19,24,25,26,27].

### 3.3. Comparative Efficacy and Safety

Recent comparative analyses have highlighted the ongoing uncertainty surrounding optimal antithrombotic management after left atrial appendage occlusion (LAAO), particularly given that post-implant strategies often diverge from the original clinical rationale for device implantation in patients with intolerance or contraindications to long-term anticoagulation [28]. In this context, the ANDES and ADALA randomized clinical trials provide important insights into early post-procedural management in device-treated patients. In ANDES, short-term full-dose oral anticoagulation was associated with a substantially lower incidence of device-related thrombosis (approximately 1–2%) compared with dual antiplatelet therapy (DAPT; approximately 6–7%), without a parallel increase in major bleeding, suggesting that transient anticoagulation may effectively mitigate early thrombotic risk during device endothelialization. Similarly, ADALA evaluated a lower-intensity strategy by comparing low-dose direct oral anticoagulation with DAPT and demonstrated complete suppression of device-related thrombosis in the anticoagulation arm, alongside a marked reduction in the composite of thromboembolic events and major bleeding (~5% vs. ~22%) [29,30].

While both ANDES and ADALA provide valuable randomized evidence in a field predominantly informed by observational studies, several limitations should be acknowledged. Both trials enrolled relatively small patient populations, thereby restricting statistical power, particularly with respect to bleeding outcomes. In addition, differences in endpoint adjudication methods and the absence of formal power calculations specifically targeting major hemorrhagic events limit the precision of safety comparisons. Accordingly, although these studies support the potential role of short-term anticoagulation strategies after LAAO, their findings should be interpreted cautiously and regarded as exploratory rather than definitive. Larger, adequately powered randomized investigations remain necessary to confirm these observations and to establish standardized post-implant therapeutic pathways.

These randomized observations are supported by a recent systematic review and meta-analysis, which reported a lower risk of device-related thrombosis with short-term anticoagulant therapy compared with antiplatelet-based regimens, without a consistent increase in major bleeding, although substantial heterogeneity and the predominance of observational data limited definitive conclusions. Importantly, across both randomized trials and pooled analyses, the apparent benefits of anticoagulation were largely confined to the early post-implant period. Taken together, these data support the concept that short-term anticoagulation—at either standard or reduced intensity—may provide superior early protection against device-related thrombosis compared with DAPT in selected LAAO recipients. Long-term management transitions toward single antiplatelet therapy or no antithrombotic treatment should remain individualized, particularly in patients with high bleeding risk [31].

Against this background, the selection of antithrombotic therapy beyond the early post-implant phase remains a critical and unresolved issue, requiring careful balancing of thrombotic protection against bleeding risk after LAAO. Mechanistic insights from a recent state-of-the-art review underscore that post-procedural risk is dynamic and closely linked to hemostatic biomarkers reflecting endothelial injury, device surface thrombogenicity, platelet activation, and patient-specific prothrombotic states. Biomarkers such as D-dimer, thrombin generation indices, and inflammatory markers have been associated with both device-related thrombosis and bleeding propensity, providing a biological rationale for tailoring antithrombotic intensity and duration rather than applying uniform regimens [32]. Within this framework, traditional warfarin-based protocols—the historical standard—effectively suppress early thrombus formation but are associated with significant bleeding risk and logistical burden, while DAPT, often used when oral anticoagulation is contraindicated, may still confer substantial bleeding liability [33].

A central issue across all therapeutic pathways is the development of device-related thrombus (DRT), which occurs in approximately 2–5% of patients, depending on surveillance protocols and device characteristics. When DRT is identified, escalation to full-dose anticoagulation—usually a DOAC or VKA—is the most common management strategy and typically results in thrombus resolution. However, DRT definitions, follow-up schedules, and treatment algorithms remain inconsistent internationally [34,35].

### 3.4. Remaining Knowledge Gaps

Despite substantial clinical experience and an expanding evidence base, several key uncertainties persist. The field lacks adequately powered randomized controlled trials comparing DOACs, VKAs, DAPT, and SAPT after LAAO, limiting the ability to define an optimal regimen [7,25,36]. Future investigations should specifically evaluate DOAC versus DAPT strategies in adequately powered multicenter randomized trials with standardized definitions of device-related thrombus and centrally adjudicated bleeding endpoints. The ideal duration of therapy remains unsettled; some studies suggest that shorter anticoagulation periods (such as 45 days) may reduce bleeding without increasing thrombotic events, but these findings require validation [35]. Randomized duration-comparison studies (e.g., 45-day versus 3-month anticoagulation) are required to determine the minimal effective treatment window.

Furthermore, currently, there is no large randomized clinical trial specifically comparing different SAPT regimens (e.g., aspirin vs. clopidogrel) after LAAO. Evidence on optimal SAPT after LAAO is limited and mostly from observational studies or propensity-matched analyses and not from dedicated randomized trials focused on SAPT alone. In patients undergoing LAAO who are unsuitable for oral anticoagulation, single antiplatelet therapy—most commonly low-dose aspirin—represents the preferred long-term therapy, with clopidogrel reserved for those with aspirin intolerance [37]. Dedicated randomized studies comparing aspirin and clopidogrel as standalone long-term strategies would clarify whether specific platelet inhibition pathways confer differential protection against late device-related thrombosis.

Additional gaps include the absence of evidence-based guidance for specific high-risk groups (e.g., patients with prior intracranial hemorrhage, severe renal dysfunction, or concurrent coronary disease requiring antiplatelets), variability in imaging protocols, and heterogeneous definitions of DRT across studies [38]. Prospective registries incorporating uniform imaging schedules (TEE and/or cardiac CT) and harmonized DRT criteria are needed to enable reliable cross-study comparisons. Moreover, device-related factors—such as design, surface characteristics, and procedural technique—may influence thrombogenicity, yet remain insufficiently characterized. Mechanistic studies integrating biomarker profiling and device-specific analyses may further refine risk stratification and support personalized post-implant management.

In summary, although temporary antithrombotic therapy after LAAO is a cornerstone of post-procedural management, consensus on the optimal regimen, duration, and follow-up strategy is still lacking. A coordinated research agenda combining randomized trials, pragmatic duration studies, and standardized imaging frameworks is essential to transition from empiric practice to evidence-driven precision therapy. Future research must prioritize randomized comparisons, standardized imaging protocols, and individualized strategies that account for each patient’s balance of thrombotic and bleeding risk [19,39,40].

The principal future research priorities across LAAO are summarized in Table 1.

## 4. Antithrombotic Therapy After Patent Foramen Ovale (PFO) Closure

ESC guidance supports percutaneous PFO closure for secondary prevention of ischemic stroke in carefully selected patients, particularly younger patients with cryptogenic stroke and high-risk PFO features, following multidisciplinary evaluation. The ESC emphasizes that closure should be considered only after exclusion of alternative stroke mechanisms, including atrial fibrillation, and in the context of shared decision-making, while acknowledging that post-closure antithrombotic therapy remains empiric and is not supported by randomized evidence [41].

**Figure 2:**
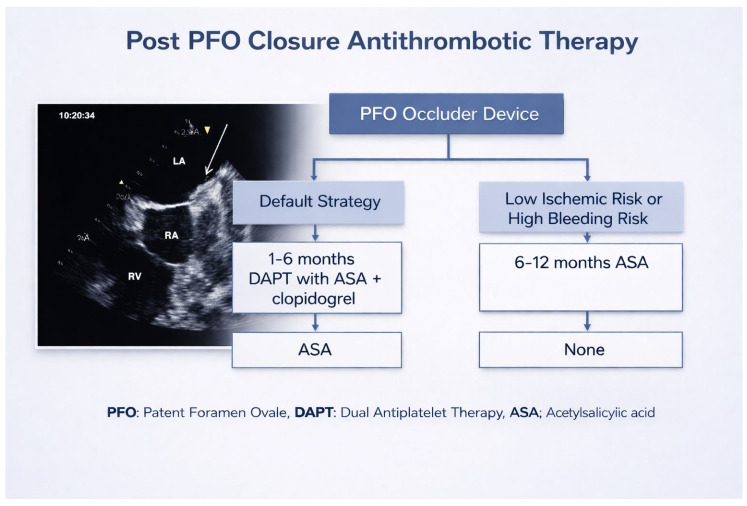
Treatment algorithm for antithrombotic therapy after PFO closure. PFO (Patent Foramen Ovale), DAPT (Dual Antiplatelet Therapy), ASA (Acetylsalicylic Acid) [TITLE: Post PFO Closure Antithrombotic Therapy].

Antithrombotic therapy following PFO closure serves two aims: to prevent early device-related thrombus and to reduce periprocedural and early embolic risk during the device endothelialization procedure. Historically, most centers prescribe a short course of dual antiplatelet therapy (DAPT) immediately after implantation (typically aspirin plus clopidogrel for 1–3 months) followed by single-agent aspirin, but practice has become more heterogeneous as new observational data and longer-term cohorts have accumulated (Figure 2) [5,42].

Comparative evidence is limited but suggestive. Large ambispective cohort data (the AIR-FORCE experience) found no significant difference in long-term clinical outcomes—including stroke, TIA, death, or major bleeding—between patients discharged on single antiplatelet therapy versus dual antiplatelet therapy, challenging the necessity of routine DAPT for every patient [5]. Similarly, long-term propensity-matched cohort data indicate that short-term antithrombotic therapy (≈6 months) after PFO closure does not increase recurrent stroke or device-thrombosis rates over extended follow-up compared with prolonged therapy, although these analyses are observational and patient selection may influence outcomes [43].

Randomized trials establishing the benefit of PFO closure over medical therapy—including RESPECT, REDUCE, CLOSE, and DEFENSE-PFO—were designed to evaluate the efficacy of mechanical closure for secondary stroke prevention rather than to compare post-procedural antithrombotic strategies. In these studies, antithrombotic therapy after device implantation functioned as protocol-mandated background care, was not randomized, and varied substantially in both composition and duration across trials, largely reflecting device-specific recommendations and evolving clinical practice. Although recurrent stroke and device-related thrombotic events were infrequent following closure, the trials were underpowered to detect differences between antithrombotic regimens, and the absence of excess events cannot be interpreted as evidence of equivalence or optimality. Moreover, concurrent improvements in device design and patient selection confound any attempt to attribute outcomes to a specific antithrombotic approach. Consequently, while these trials support the safety of short-term antiplatelet therapy after PFO closure, they do not provide a robust evidence base to define the optimal type or duration of post-closure antithrombotic therapy, leaving current practice dependent on expert consensus rather than randomized data [44,45,46,47,48,49,50].

In the three randomized trials establishing the efficacy of PFO closure, detailed percentage-based reporting for post-procedural antithrombotic therapy was limited. In RESPECT, short-term dual antiplatelet therapy followed by aspirin monotherapy was mandated after device implantation, but adherence and regimen-specific percentages were not reported. In REDUCE, antiplatelet therapy was required for all participants and anticoagulation was excluded, allowing the statement that approximately 100% of patients received antiplatelet therapy, although no percentages for specific agents or duration of dual therapy were provided. CLOSE uniquely included a randomized comparison with anticoagulation, in which approximately 93% of patients received vitamin K antagonists; however, as in the other trials, no percentage breakdown of antiplatelet regimens was reported for patients undergoing PFO closure. Consequently, while broad treatment constraints can be described, none of these trials provided reliable data to define optimal post-closure antithrombotic practice.

Key clinical considerations include patient age, thrombophilia status, concurrent indications for antiplatelets or anticoagulation, and device type. In patients with documented thrombophilia or other unusual prothrombotic states, individualized strategies (often involving longer or different antithrombotic regimens) are frequently applied, but robust evidence to guide these choices is lacking [47]. In summary, while antiplatelet therapy after PFO closure is widely used, the optimal agent(s) and duration remain unsettled, and decisions should be individualized pending randomized data that directly compare regimens [5,43].

Observed differences in antiplatelet regimens, after implantation of devices, are attributable to protocol-driven practice and manufacturer recommendations, without randomized evidence to support device-specific outcome optimization. For the Amplatzer PFO Occluder, shorter dual antiplatelet therapy—typically about one month followed by aspirin monotherapy—became standard, consistent with the RESPECT protocol and assumptions of relatively rapid endothelialization. In contrast, Gore Cardioform (and earlier Helex) devices have generally been associated with longer dual antiplatelet therapy, often three to six months, reflecting early concerns about device surface characteristics and endothelialization as incorporated into the REDUCE protocol and instructions for use. Importantly, these differences are not supported by randomized data demonstrating device-specific differences in thrombotic or ischemic outcomes and should be viewed as empiric risk-mitigation strategies rather than evidence-based optimization [51,52,53].

## 5. Antithrombotic Therapy After Secundum Atrial Septal Defect (ASD) Closure

**Figure 3:**
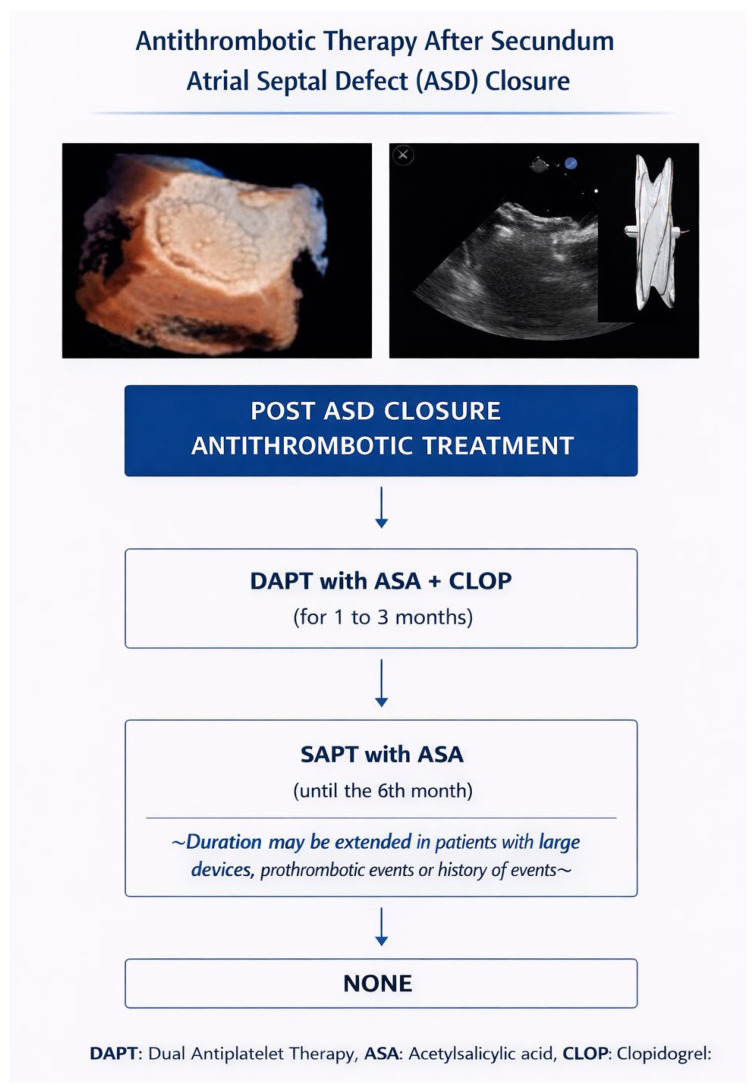
Treatment algorithm for antithrombotic therapy after ASD closure. DAPT (Dual Antiplatelet Therapy), ASA (Acetylsalicylic Acid), CLOP (Clopidogrel) [TITLE: Antithrombotic Therapy After Secundum Atrial Septal Defect (ASD) Closure].

ESC guidelines recommend closure of a secundum atrial septal defect in the presence of a significant left-to-right shunt with right-sided volume overload, favoring percutaneous device closure when anatomically suitable and contraindicating intervention in patients with severe irreversible pulmonary hypertension (Class I, Level of Evidence B) [41]. Post-device antithrombotic management aims principally to prevent thrombus formation on the device surface during the endothelialization period and to minimize early embolic complications. Contemporary practice almost uniformly prescribes antiplatelet therapy following ASD device deployment—most commonly aspirin for approximately six months, often with a short initial course of clopidogrel (e.g., 1 month) in some centers—reflecting guideline recommendations and angioscopic evidence of device neo-endothelialization around 6 months (Figure 3) [54].

The supporting evidence is largely observational and physiologic: angioscopy and imaging studies demonstrate that neo-endothelial coverage of septal occluder devices is typically incomplete in the very early months and improves by the 6-month mark, providing a biologic rationale for a 6-month aspirin regimen [54]. Comparative trials are scarce; smaller randomized work (e.g., investigations of antiplatelet strategies for symptom endpoints such as migraine after ASD closure) and registry data report low rates of device thrombus and thromboembolic events when aspirin (with or without a brief DAPT period) is used, supporting the safety of this common approach [47].

Despite this consensus practice, unresolved issues remain: the optimal duration of clopidogrel when used (1 vs. 3 months), the necessity of DAPT in older adults or patients with large devices, and management of patients with prothrombotic disorders. Additionally, rare reports of late device-related thrombus or infective complications underscore the need for appropriate follow-up and for studies that correlate device type and deployment technique with thrombotic risk [54]. Overall, current evidence supports aspirin for approximately 6 months after ASD device closure, with short DAPT reserved for specific clinical scenarios, but more comparative data would refine these recommendations [47,54].

The principal clinical trials evaluating post-procedural antithrombotic strategies across LAAO and PFO closure are summarized in Table 2.

## 6. Periprocedural Antithrombotic Therapy in Atrial Structural Interventions

In randomized and large prospective studies of PFO, ASD, and LAAO, intraprocedural anticoagulation with unfractionated heparin is uniformly employed to prevent catheter- and device-related thrombosis. In the pivotal PFO closure trials—RESPECT, REDUCE, and CLOSE—administration of intravenous heparin during the procedure was mandated, typically targeting an activated clotting time above procedural thresholds, although exact dosing and targets were not consistently reported. Patients were generally receiving antiplatelet therapy at the time of intervention; however, none of these trials required or systematically reported standardized loading doses of aspirin or clopidogrel immediately before device implantation, leaving antiplatelet loading to operator discretion and local practice. Similarly, ASD closure studies follow analogous periprocedural heparinization strategies without standardized antiplatelet loading requirements. In contrast, LAAO trials and registries uniformly mandate intraprocedural heparinization and more explicitly document periprocedural antithrombotic preparation, although routine clopidogrel or aspirin loading prior to implantation is variably applied and not universally required. Overall, while periprocedural heparin use is consistent across all three interventions, standardized reporting of antiplatelet loading strategies is limited, reflecting their treatment as background procedural care rather than a formally studied intervention [41,44,45,46,55,56,57].

## 7. Discussion

The management of antithrombotic therapy following LAAO, PFO closure, and ASD closure reflects three distinct clinical landscapes, each shaped by different procedural indications, patient comorbidities, and device-specific thrombogenicity. When these interventions are considered together, a central finding emerges: although antithrombotic therapy is universally required to support device endothelialization and prevent device-related thrombus, the optimal regimen remains poorly defined, with substantial variability in clinical practice driven more by convention than by high-grade evidence.

One of the most striking contrasts lies between the high-risk, often elderly LAAO population and the relatively young, low-risk patients undergoing PFO or ASD closure. In this context, young patients participating in competitive sports who undergo PFO or ASD closure may require careful reassessment or potential de-escalation of post-procedural antiplatelet therapy to mitigate undesirable bleeding risk, particularly in contact or high-intensity disciplines, an issue that remains insufficiently studied and warrants dedicated future investigation [58]. In LAAO, the residual surface exposure of the metallic device and the prothrombotic substrate of atrial fibrillation sustain a measurable risk of device-related thrombus, highlighted in contemporary imaging registries [20]. This has prompted the adoption of short-term anticoagulant-based regimens, including growing interest in DOACs, supported by analyses suggesting favorable bleeding profiles without loss of efficacy. In contrast, septal closure devices used for PFO and ASD are associated with significantly lower thrombotic potential, which may explain why antiplatelet-only regimens appear sufficient, as suggested by observational evidence for simplified approaches after PFO closure [5,43]. These divergent therapeutic philosophies underscore how baseline patient risk, rather than device type alone, drives the intensity of pharmacologic prevention.

In this context, reported device-related thrombosis (DRT) after PFO closure is rare and inconsistently documented. In extended follow-up from RESPECT, no device thrombus was reported with the Amplatzer occluder, whereas REDUCE and CLOSE did not provide trial-level DRT percentages in their primary safety analyses, reflecting both the low incidence of events and the absence of systematic reporting. Earlier experience with the STARFlex device in the CLOSURE I era demonstrated higher thrombotic signals (~1.1%), although this device is no longer in routine use. In contrast, DRT after left atrial appendage occlusion (LAAO) is reported more frequently, with pooled trial analyses and regulatory datasets describing rates in the range of approximately 1–4%, and some device- and modality-specific series suggesting lower rates with Amulet compared with Watchman. However, interpretation of these differences is complicated by the lack of standardization in postprocedural surveillance: DRT detection after LAAO is highly dependent on the frequency and modality of follow-up imaging, and heterogeneous echocardiographic and CT protocols likely account for much of the variability in published rates. By comparison, routine postprocedural imaging after PFO and ASD closure is far less standardized, reflecting lower perceived thrombotic risk but also potentially obscuring subclinical thrombus formation. Until follow-up strategies and imaging protocols become more uniform across studies, it will remain difficult to determine whether observed differences in thrombotic risk reflect intrinsic device properties or simply differences in detection practices [44,45,46,59,60,61].

Beyond differences in patient characteristics and surveillance protocols, procedural and device-related determinants likely contribute substantially to the heterogeneity in thrombotic risk observed after atrial closure interventions. Suboptimal device positioning, inadequate appendage sealing, or significant peridevice leaks may create regions of residual flow and localized stasis, thereby promoting thrombus formation on exposed device surfaces, particularly during the early endothelialization phase. Recent data further suggest that excessive device compression may also influence post-implant outcomes, underscoring the importance of balanced deployment and optimal sizing. These technical factors underscore that device-related thrombosis is not solely a function of systemic prothrombotic burden but may also reflect procedural precision. Consequently, meticulous intraprocedural imaging, confirmation of appropriate device compression and sealing, and careful post-implant assessment are critical components of risk mitigation and may meaningfully influence subsequent antithrombotic strategy [59,62,63,64,65].

An additional consideration following septal closure interventions is the documented incidence of new-onset atrial fibrillation after both PFO and ASD device implantation. Although frequently transient and occurring early after the procedure (3–6% of patients), AF episodes (approximately 0.5–1%) may persist or recur and thereby modify the long-term thromboembolic risk profile of otherwise low-risk patients. This evolving arrhythmic substrate introduces complexity into antithrombotic decision-making, as individuals initially treated with antiplatelet-based strategies may subsequently meet criteria for oral anticoagulation if sustained atrial fibrillation is confirmed. Consequently, rhythm surveillance and reassessment of thrombotic risk over time represent important components of post-closure management [44].

Across interventions, there is growing recognition that individual risk stratification should guide antithrombotic duration more than device or procedure type. Factors such as thrombophilia, renal impairment, prior major bleeding, atrial arrhythmias, or concomitant need for antiplatelet therapy create overlapping risk profiles that challenge the utility of fixed, protocolized regimens. Evidence from PFO closure studies, which show that shorter antithrombotic courses do not worsen outcomes [2,43], suggests that dynamic, risk-adapted strategies may be both effective and safe. However, such approaches require stronger validation before they can be broadly implemented.

Finally, a major knowledge gap persists in understanding how device engineering, including structural materials, surface textures, and polymer coatings, modulates thrombogenicity. Laboratory and early clinical observations suggest that modern devices may achieve faster endothelial coverage; however, these findings have yet to be translated into therapeutic de-escalation algorithms [66]. As next-generation occluders continue to evolve, the relationship between device design and the need for prolonged antithrombotic therapy will likely become an increasingly important research focus.

In aggregate, the available evidence suggests that while current antithrombotic strategies are generally effective, they may be more intensive or prolonged than necessary for many patients, particularly those undergoing PFO or ASD closure. The field now requires rigorously designed randomized trials, consistent imaging definitions, and device-specific and patient-specific risk models. Such advances will be essential to establish a more precise, individualized framework capable of reducing thrombotic events without exposing patients to avoidable bleeding risk [67].

## 8. Conclusions

Patient-specific risk rather than uniform protocols should guide antithrombotic therapy following LAAO, PFO, and ASD closure. Current evidence supports streamlined regimens for PFO and ASD devices, whereas LAAO still demands structured antithrombotic therapy and routine imaging to prevent device-related thrombus. Despite growing clinical experience, optimal strategies remain uncertain, and practice patterns vary widely. Clear, comparative trials are now essential to refine therapy and ensure safer, more consistent postprocedural care across structural heart interventions.

## Figures and Tables

**Table 1:** Proposed Research Agenda for Antithrombotic Therapy After LAAO.

Research Domain	Unresolved Clinical Question	Recommended Study Design	Primary Endpoints
Post-LAAO regimen comparison	Does DOAC therapy provide superior efficacy and safety compared with DAPT after LAAO?	Multicenter randomized controlled trial with centralized event adjudication	Device-related thrombus (DRT), stroke/systemic embolism, major bleeding
Anticoagulation duration	What is the minimal effective duration of anticoagulation (e.g., 45 days vs. 3 months)?	Randomized duration-comparison trial	DRT incidence, thromboembolic events, major bleeding
SAPT optimization	Is aspirin superior or equivalent to clopidogrel as long-term SAPT after LAAO?	Dedicated randomized controlled trial	Late DRT, ischemic stroke, bleeding complications
Imaging standardization	What is the optimal surveillance modality and timing for DRT detection?	Prospective registry with predefined imaging protocol (TEE and/or CT)	DRT detection rate, inter-modality agreement
High-risk subgroups	How should therapy be tailored in patients with intracranial hemorrhage, renal dysfunction, or concomitant CAD?	Stratified prospective cohort or pragmatic RCT	Net clinical benefit (ischemic + bleeding outcomes)
Mechanistic stratification	Can biomarkers or device-specific characteristics predict thrombotic risk?	Translational mechanistic studies with biomarker profiling	Correlation of biomarkers with DRT and bleeding

Abbreviations: DOAC, direct oral anticoagulant; DAPT, dual antiplatelet therapy; SAPT, single antiplatelet therapy; LAAO, left atrial appendage occlusion; TEE, transesophageal echocardiography; CT, computed tomography; CAD, coronary artery disease; RCT, randomized controlled trial.

**Table 2:** Key Trials Evaluating Antithrombotic Strategies After LAAO and PFO Closure.

Study	Population	Antithrombotic Comparison	Sample Size	Main Findings	Clinical Implication
ANDES	LAAO recipients	DOAC vs. DAPT	350	Lower DRT incidence with DOAC; no significant increase in major bleeding	Supports short-term anticoagulation after LAAO
ADALA	LAAO recipients	Low-dose DOAC vs. DAPT	155	Suppression of DRT; reduced composite thromboembolic/bleeding endpoint	Suggests the feasibility of reduced-intensity DOAC
RESPECT	PFO closure	Closure + antiplatelet vs. medical therapy	980	Reduced recurrent ischemic stroke; AF ~3–6% (mostly early)	Established benefit of closure; transient AF common
REDUCE	PFO closure	Closure + antiplatelet vs. antiplatelet alone	664	Lower recurrent stroke; AF ~6% early post-procedure	Reinforces closure efficacy; AF consideration relevant

Abbreviations: AF, atrial fibrillation; DAPT, dual antiplatelet therapy; DOAC, direct oral anticoagulant; DRT, device-related thrombus; LAAO, left atrial appendage occlusion; PFO, patent foramen ovale.

## Data Availability

No new data were created or analyzed in this study. Data sharing is not applicable to this article.

## Abbreviations

The following abbreviations are used in this manuscript:

LAAO	Left Atrial Appendage Occlusion
PFO	Patent Foramen Ovale
ASD	Atrial Septal Defect
DOAC	Direct Oral Anticoagulants
SAPT	Single Antiplatelet Therapy
DAPT	Dual Antiplatelet Therapy
LAAC	Left Atrial Appendage Closure
DRT	Device-Related Thrombosis
OAC	Oral Anticoagulants
VKA	Vitamin K Antagonists
ESC	European Society of Cardiology
TIA	Transient Ischemic Attack
